# Chromosomal polymorphisms associated with reproductive outcomes after IVF-ET

**DOI:** 10.1007/s10815-020-01793-8

**Published:** 2020-05-25

**Authors:** Sai-jiao Li, Yan-xiang Cheng, Ye-Shang, Dan-ni Zhou, Yin Zhang, Tai-lang Yin, Jing Yang

**Affiliations:** 1 Reproductive Medical Center, Renmin Hospital of Wuhan University, No. 238 JieFang Road, Wuhan, 430060 People’s Republic of China; 2 Hubei Clinic Research Center for Assisted Reproductive Technology and Embryonic Development, No. 238 JieFang Road, Wuhan, 430060 People’s Republic of China; 3 Gynecology Department, Renmin Hospital of Wuhan University, No. 238 JieFang Road, Wuhan, 430060 People’s Republic of China

**Keywords:** Chromosomal polymorphism, IVF-ET, inv(9), Miscarriage

## Abstract

**Purpose:**

This study aimed to investigate the effect of the detail type of chromosomal polymorphisms (1/9/16qh^+/−^, D/G group polymorphisms, and inv(9)) on the IVF-ET outcomes.

**Methods:**

A total of 1335 infertile couples undergoing IVF/ICSI were enrolled and comprehensively analyzed the correlation between three detail types of chromosomal polymorphisms (1/9/16qh^+/−^, D/G group polymorphisms, and inv(9)) and the outcome of IVF/ICSI embryo transfer. The fertilized rate, cleaved embryo rate, good-quality embryo rate, clinical pregnancy rate, implantation rate, and early stage miscarriage rate were compared between the chromosomal polymorphisms groups and the control group.

**Results:**

Both the inv(9) and D/G group chromosomal polymorphisms related to female infertility significantly lead to a lower 2PN cleavage rate (86.44% vs. 97.58% and 90.67% vs. 97.58%, respectively, *P* < 0.05) undergoing IVF insemination, the inv(9) adversely increasing the early miscarriage rate, either undergoing IVF (21.4% vs. 3.0%, *P* < 0.05) or ICSI (50.0% vs. 2.0%, *P* < 0.05) insemination, female carriers (23.08% vs. 2.87%, *P* < 0.05) or male carriers (44.44% vs. 2.87%, *P* < 0.05). For D/G groups, ICSI insemination may increase the implantation rate (44.8% vs. 23.69%, *P* < 0.05) and clinical pregnancy rate (78.6% vs. 40.65%, *P* < 0.05). 1/9/16qh^+/−^ had no apparent adverse effect on the patient’s clinical outcomes.

**Conclusions:**

Our study suggests that chromosome karyotype analysis is necessary for IVF patients in clinical practice; we should afford individual genetic counseling suggestion according to the polymorphism types.

## Introduction

Chromosomal polymorphisms mainly refer to variants in the chromosomal heterochromatin region. In routine cytogenetics, increases or decreases in the lengths of the heterochromatic regions on the long arms of these chromosomes are designated as 1qh^+/−^, 9qh^+/−^, 16qh^+/−^, and Yqh^+/−^. Findings regarding these regions are prevalent, and the frequencies of 9qh^+^ and Yqh^+^ have been reported to be approximately 2.44% and 2.85%, respectively [1]. However, more earlier reports presented a higher incidence of approximately 7.60% for 9qh^+^ polymorphisms [2, 3]. An increase or decrease in the length of the short arm of acrocentric (acro) D-genome and G-genome (D/G) group chromosomes is designated p±, while an increase or decrease in the length of short arm satellites and stalks is designated ps^+/−^ and pstk^+/−^, respectively [4, 5]. Variants in the D/G group have been reported to constitute approximately 3.96% of variants (1). Pericentric inversion of chromosome 9—regularly referred to as inversion 9 (inv(9))—is one of the most common variations in the human karyotype; the estimated frequency varies from 1 to 4% in extensive epidemiological studies [6–11]. The latest version of the International System for Cytogenetic Nomenclature (ISCN) [12] refers to inv(9) (p12q13) as a chromosomal polymorphism (or generally heteromorphism) with no clinical significance.

For a long time, constitutive heterochromatin has been categorized as minor chromosomal rearrangements, which are formed by tandemly organized, highly repeated sequences of satellite DNA that have no apparent coding potential and do not correlate with abnormal phenotypes [13, 14]. However, an increasing number of studies have reported the potential effects of chromosomal polymorphisms on reproductive capacity.

In recent years, a growing number of studies have reported an increased incidence of chromosomal polymorphism variations in infertile couples [15–18], in patients with spontaneous miscarriages [19–22] and even in patients with psychiatric disorders [23]. However, how chromosomal polymorphisms affect fertility remains unclear. Very few reports had concern the impact of chromosomal polymorphic variations on assisted reproductive technology (ART) outcomes [24–27]. Besides, most of the studies just focus on the ART treatment outcomes of infertile males with Y chromosomal variations [25–28]; only a few studies had especially paid attention to the ART outcomes of the female with chromosomal polymorphisms. Moreover, the majority of the previous reports simply combined all chromosomal heteromorphism types into one group to study their effects on fertility [29, 30]. No study, however, has stratified infertility according to the specific type of chromosomal polymorphism, other than Y chromosome variations, on infertility treatments, including IVF-embryo transfer.

Therefore, this retrospective study comprehensively analyzed the correlations between chromosomal polymorphisms, which were subdivided into three groups (the 1/9/16qh^+/−^ group, D/G group, and inv(9) group), and the outcome of IVF-embryo transfer in infertile couples.

## Materials and methods

### Participants

A retrospective, single-center cohort study was conducted between October 2014 and November 2017 at the Reproductive Medicine Center in Renmin Hospital of Wuhan University in Wuhan, China. Infertile couples who had received their first IVF-embryo transfer treatment cycle and carried out karyotype analyses were included.

### Chromosome karyotype analysis

G-banded chromosome karyotype analysis of cultured peripheral blood lymphocytes was carried out for all ART couples. At least 20 metaphases were analyzed, and five metaphases were karyotyped for each case. The banding resolution was 400–550 bands per haploid set (BPHS). Two independent researchers confirmed all slides, and the results were reported according to the ISCN 2009 after selective banding studies. C-banding and nucleolar organizing region (NOR) banding were conducted to assist the karyotype analysis. Distinct polymorphic variants of the lengths of the stalks (pstk) and the size of the satellites (ps) of the acro chromosomes were documented, and the variant was at least twice the size of its corresponding region on the other homolog. Polymorphic variations in centromeric heterochromatin length on the long arms of chromosomes 1, 9, and 16 (1qh, 9qh, and 16qh) were also recorded. Only consistent and very prominent polymorphisms were reported; additionally, it was noted that these were normal variants.

### Study design

Infertile couples were divided into four groups according to the karyotype analysis results: 129 couples with 1qh^+/−^, 9qh^+/−^, or 16qh^+/−^ (1/9/16qh^+/−^ group); 55 couples with ps^+/−^, pss, or pstk^+/−^ of the variants in the NOR of the acro chromosomes (D/G group); 62 couples with pericentric inversion of chromosomes 9 (inv(9) group) and 1088 couples with normal chromosomes (control group) in which the etiology of infertility was the female tubal factor. The frequency of chromosomal polymorphic variations was calculated. The exclusion criteria were as follows: the female reproductive age ≥ 38 years, body mass index (BMI) < 18 or > 25 kg/m^2^, day 3 FSH concentration > 10 IU/L, ovulation dysfunction (such as polycystic ovarian syndrome) or female with endocrine disorders (diabetes mellitus, thyroid dysfunction, hyperprolactinemia, congenital adrenal hyperplasia, Cushing syndrome), or uterine anomaly confirmed by either hysterosalpingography or hysteroscopy. Couples with polymorphic variants of chromosomes in both males and females were excluded from the study.

### In vitro fertilization/intracytoplasmic sperm injection procedure

The patients underwent ovarian stimulation accomplished with a GnRH antagonist protocol or long luteal downregulation protocol. All patients had a baseline transvaginal ultrasound on menstrual cycle day 2 or day 3. Patient response was monitored with transvaginal ultrasounds for follicular measurements and serum estradiol (E_2_) levels. When three follicles diameter reached ≥ 17 mm, patients were triggered with 10,000 IU HCG intramuscularly. Oocyte retrieval was performed 36 h later. Intracytoplasmic sperm injection (ICSI) was performed if the concentration of motile sperm was < 1 × 10^6^/mL after sperm preparation on the day of oocyte retrieval; otherwise, a conventional IVF method was used. Embryo transfers were performed 72 h after oocyte retrieval.

Monitoring of patients included general information, symptoms, embryonic condition, BMI, and other independent variables consisted of female age, IVF or ICSI, female basal FSH, the protocol of ovarian stimulation, the dosage of gonadotrophin (Gn), the sex hormone level on the day of HCG injected, the thickness of endometrium on HCG day, and the oocytes retrieved number. The fertilized rate, 2 pronuclei (2PN) cleaved rate, good-quality embryo rate, clinical pregnancy rate (CPR, gestational sac seen at 6.5 weeks), implantation rate (number of gestational sacs seen at 6.5 weeks per number of embryos transferred), and early stage miscarriage rate were calculated and compared between the four groups.

### Statistical analysis

All statistical analyses were performed with the SPSS 24.0 statistical software (Chicago, IL, USA) according to the intention to treat principle. Data were presented as mean ± SD. Chi-square or Fisher exact tests were used for categorical *l* variables. ANOVA were performed on comparison among multiple groups. A significant result means that the *P* value for the ordinal level measure is < 0.05, and the confidence interval (CI) is 95%.

## Results

As shown in Table 1, 246 out of 1335 couples carried chromosomal polymorphisms: the male carriers accounted for 53.25% (131/246), and female carriers accounted for 46.75% (115/246). In males, the Yqh^+/−^ polymorphisms were not present, the most frequent polymorphism type was 1qh^+/−^ (32.06%), and the least frequent polymorphism type was 15pstk^+/−^ and 13ps^+/−^ (0.76%). The most and least frequent types in females were inv(9) (p13q21) (32.17%) and 13ps^+/−^, 14ps^+/−^, 13pstk^+/−^, and 14pstk^+/−^ (0.87%), respectively.

**Table 1:** Frequency and polymorphic variants observed in chromosomal polymorphisms patients

Classification	Karyotypes	No. of male (*n* = 131)	Composition ratio (%)	No. of female (*n* = 115)	Composition ratio (%)	Total no. (*n* = 246)	Composition ratio (%)
qh^+^							
	1qh^+/−^	42	32.06	32	27.83	74	30.08
	9qh^+/−^	14	10.69	7	6.09	21	8.53
	16qh^+/−^	19	14.50	15	13.04	34	13.82
Chromosome variation in D/G genomes							
	13ps^+/−^	1	0.76	1	0.87	2	0.81
	14ps^+/−^	2	1.53	1	0.87	3	1.22
	15ps^+/−^/pss	3	2.29	2	1.74	5	2.03
	21ps^+/−^/pss	4	3.05	5	4.34	9	3.66
	22ps^+/−^	2	1.53	3	2.61	5	2.03
	13pstk^+/−^	3	2.29	1	0.87	4	1.63
	14pstk^+/−^	2	1.53	1	0.87	3	1.22
	15pstk^+/−^	1	0.76	4	3.48	5	2.03
	21pstk^+/−^	6	4.58	2	1.74	8	3.25
	22pstk^+/−^	7	5.34	4	3.48	11	4.29
Chromosome 9 pericentric inversion							
	inv(9) (p13q21)	25	19.08	37	32.17	62	25.20

Values are number (or percentage)

*qh*^*+/−*^ increases or decrease in the lengths of the heterochromatic regions on the long arms of chromosomes, *ps*^*+/−*^ increase or decrease in the length of short arm satellites, *pss* two short arm satellites, *pstk*^*+*^ increase or decrease in the length of short arm stalks, *inv(9)* inversion 9

As shown in Table 2, the four groups were compared in terms of the age, BMI, infertility duration, baseline FSH level, duration of Gn stimulation, the total Gn dose, the E_2_ concentration on the day of HCG administration, the retrieved oocyte number, progesterone level, and endometrial thickness on the day of HCG administration. Except for the IVF and ICSI percentage, there were no significant differences for any of the parameters (*P* > 0.05).

**Table 2:** Basal characteristics of infertile couples

	Control	1, 9, 16qh^+/−^ group	D/G group	inv(9)	*P* value
No. of case	1088	129	56	62	
Female age (years)	30.88 ± 3.23	31.35 ± 4.43	30.91 ± 4.03	31.04 ± 4.56	0.799
BMI of women (kg/m^2^)	22.39 ± 3.16	22.05 ± 3.61	22.65 ± 3.50	22.76 ± 3.56	0.509
Duration of infertility (years)	4.3 ± 3.09	5.14 ± 3.66	4.75 ± 2.87	4.78 ± 3.37	0.803
Baseline FSH (IU/L)	6.68 ± 1.79	6.63 ± 2.13	6.52 ± 1.61	6.73 ± 1.81	0.159
Male carrier (%)	-	74 (57.4)	32 (57.1)	25 (40.3)	0.068
Female carrier (%)	-	55 (42.6)	24 (42.9)	37 (59.7)	
Long protocol (%)	914(77.33)	98 (75.97)	41 (73.21)	46 (74.19)	0.838
GnRH antagonist protocol (%)	268(22.67)	31 (24.03)	15 (26.79)	16 (25.81)	
IVF (%)	908(83.5)	90 (69.8)	34 (60.7)	44 (71.0)	0.014
ICSI (%)	180(16.5)	39 (30.2)	22 (39.3)	18 (29.0)	
Duration of Gn (days)	10.25 ± 1.82	10.13 ± 1.65	10.38 ± 1.64	10.50 ± 2.69	0.142
Starting Gn dose (IU)	192.89 ± 38.98	196.97 ± 45.21	194.53 ± 41.89	196.97 ± 45.22	0.360
Total Gn ampoules(75 IU)	28.69 ± 9.98	29.00 ± 10.76	27.92 ± 8.89	30.32 ± 14.22	0.355
E_2_ level on HCG day (pg/mL)	4286.67 ± 2202.32	3899.19 ± 2204.88	4421.89 ± 2748.18	3988.46 ± 2417.62	0.139
Thickness of endometrium on HCG day (mm)	1.05 ± 0.21	1.03 ± 0.21	1.09 ± 0.22	1.06 ± 0.21	0.206
Progesterone	1.14 ± 0.57	1.10 ± 0.51	1.05 ± 0.45	1.13 ± 0.53	0.206

Values are number (or percentage). *P* value < 0.05 was considered to be significantly. All continuous variables are expressed as mean ± SD

*BMI* body mass index, *FSH* follicle stimulating hormone, *GnRH* gonadotropin-releasing hormone, *IVF* in vitro fertilization, *ICSI* intracytoplasmic sperm injection, *Gn* gonadotropin, *HCG* human chorionic gonadotropin, *E*_*2*_ estradiol

To determine whether the insemination methods affect the clinical outcomes of chromosomal polymorphisms patients, we studied the embryological and clinical outcomes of chromosomal polymorphism carriers undergoing IVF or ICSI, respectively, as shown in Table 3 and Table 4.

**Table 3:** Comparison of clinical outcomes of IVF-ET cycles among the four groups

	Control	1, 9, 16qh^+/−^ group	D/G group	inv(9)
No. of case	908	90	33	44
No. oocytes retrieved	13.67 ± 5.87	12.41 ± 6.93	13.88 ± 7.83	13.59 ± 8.39
1PN fertilization rate (%)	3.45 ± 6.01	4.40 ± 1.25	2.07 ± 3.99	1.71 ± 7.71
2PN fertilization rate (%)	56.39 ± 19.46	57.19 ± 23.44	57.41 ± 23.92	57.49 ± 27.33
Multi-PN fertilization rate (%)	9.73 ± 10.75	9.07 ± 10.78	10.28 ± 16.01	5.49 ± 8.09
Fertilization rate (%)	69.58 ± 19.44	70.44 ± 21.03	67.59 ± 25.52	64.70 ± 26.80
2PN cleavage rate (%)	97.66 ± 7.15	96.36 ± 16.07	92.59 ± 23.84^a^	88.58 ± 29.17^a^
Quality embryo rate (%)	54.48 ± 24.92	53.04 ± 27.99	53.74 ± 26.23	55.69 ± 32.93
Implantation rate (%)	32.4 (394/1215)	30.4 (38/125)	30.8 (12/39)	37.5 (18/48)
Clinical pregnancy rate (%)	47.3 (298/630)	47.0 (31/66)	45 (9/20)	58.3 (14/24)
Early miscarriage rate (%)	3.0 (9/298)	3.2 (1/31)*	0 (0/9)	21.4 (3/14)^b^*

^a^*P* < 0.05, difference between chromosomal polymorphisms group and the control group

^b^*P* < 0.01, difference between chromosomal polymorphisms group and the control group

*PN* pronuclei

*Fisher’s exact test

All continuous variables are expressed as mean ± SD

**Table 4:** Comparison of the outcomes of ICSI-ET cycles among the four groups

	Control	1, 9, 16qh^+/−^ group	D/G group	inv(9)
No. of case	180	39	22	18
No. oocytes retrieved	12.93 ± 5.61	14.31 ± 6.69	15.91 ± 6.54	13.56 ± 8.34
1PN fertilization rate	2.49 ± 4.82	3.37 ± 6.81	1.76 ± 3.72	5.53 ± 6.32
2PN fertilization rate	54.03 ± 20.84	55.45 ± 19.63	53.93 ± 20.07	56.13 ± 23.97
Multi-PN fertilization rate	1.87 ± 4.84	1.92 ± 3.64	1.45 ± 3.44	1.34 ± 3.63
Fertilization rate (%)	59.07 ± 19.02	60.74 ± 19.53	57.13 ± 19.24	63.00 ± 22.82
2PN cleavage rate (%)	97.16 ± 11.52	95.11 ± 17.93	98.46 ± 4.24	93.03 ± 23.45
Quality embryo rate (%)	52.33 ± 28.15	57.18 ± 32.22	59.27 ± 18.34	50.46 ± 28.69
Implantation rate (%)	23.69 (59/190)	28.6 (16/56)	44.8 (13/29)^a^	30.8 (8/26)
Clinical pregnancy rate (%)	40.65 (50/123)	37.9 (11/29)	78.6 (11/14)^a^	61.5 (8/13)
Early miscarriage rate (%)	2 (1/50)*	9.1 (1/11)*	0 (0/11)	50 (4/8)^b^*

^a^*P* value < 0.05, difference between chromosomal polymorphisms group and control group

^b^*P* value < 0.01, difference between chromosomal polymorphisms group and control group

*PN* pronuclei

*Fisher’s exact test

All continuous variables are expressed as mean ± SD

When the analyses were limited to patients undergoing IVF insemination, as shown in Table 3, the results indicated that there were no significant differences in the fertilization rate, 1PN fertilization rate, 2PN fertilization rate, multi-PN fertilization rate, good-quality embryo rate, clinical pregnancy rate, or implantation rate (*P* > 0.05). However, the D/G group and inv(9) group had lower 2PN cleavage rates than the control group (92.59% and 88.58% vs. 97.66%, respectively) (*P* < 0.05), and the early miscarriage rate in the inv(9) patients was higher than that in the control group (21.4% vs. 3.0%, *P* < 0.05).

Similarly, we further restricted the analysis to the ICSI patients, and the results indicated that there were no significant differences in the fertilization rate, 1PN fertilization rate, 2PN fertilization rate, multi-PN fertilization rate, good-quality embryo rate, or implantation rate among the four groups. Surprisingly, the clinical pregnancy rate of the D/G group was significantly higher than that of the control group, and the early miscarriage rate in the inv(9) group, similar to that in the IVF patients, was also significantly higher than that in the control group (Table 4).

To further determine whether the carriers’ gender affect the IVF/ICSI outcomes in chromosomal polymorphism patients, we compared the embryological and clinical outcomes of the female and male carriers, as shown in Table 5. The results indicated that the fertilization rate and 2PN cleavage rate of female carriers in the D/G group and inv(9) were significantly lower than those in the control group. Interestingly, the couples with male carriers in these two groups did not show any significant differences. In addition, both the female and male carriers in the inv(9) group had a higher early miscarriage rate than the control group, but there were no significant differences in the other groups (Table 5).

**Table 5:** Comparison of the IVF/ICSI outcomes among the four groups

	Control	1, 9, 16qh^+/−^ group		D/G group		inv(9) group	
		Female	Male	Female	Male	Female	Male
No. of case	1088	54	75	24	31	37	25
No. oocytes retrieved	13.55 ± 5.83	12.27 ± 6.77	13.51 ± 6.98	15.08 ± 7.92	14.37 ± 7.01	13.73 ± 7.65	13.36 ± 9.36
1PN fertilization rate (%)	3.29 ± 5.84	3.53 ± 6.28	4.50 ± 13.70	2.04 ± 3.71	1.88 ± 4.03	4.35 ± 9.30	0.56 ± 1.94^ab^
2PN fertilization rate (%)	56.00 ± 19.71	57.09 ± 21.28	56.34 ± 23.16	49.72 ± 24.8^b^	60.7 ± 19.38	53.51 ± 28.19	62.39 ± 22.47
Multi-PN fertilization rate (%)	8.43 ± 10.04	7.07 ± 11.27	6.79 ± 8.59	3.25 ± 10.31^ab^	9.49 ± 14.78	3.51 ± 6.60^a^	5.45 ± 8.24
Fertilization rate (%)	67.73 ± 20.08	67.34 ± 20.74	67.63 ± 21.32	55.01 ± 27.43^ab^	69.84 ± 18.32	61.37 ± 27.89^a^	68.40 ± 21.42
2PN cleavage rate (%)	97.58 ± 8.03	96.54 ± 15.15	95.57 ± 17.68	90.67 ± 28.16^ab^	98.06 ± 4.42	86.44 ± 31.40^ab^	94.95 ± 19.99
Quality embryo rate (%)	54.02 ± 25.63	53.05 ± 29.745	55.22 ± 29.09	55.68 ± 23.66	56.09 ± 23.63	54.88 ± 34.46	53.13 ± 22.52
Implantation rate (%)	32.24 (453/1405)	33.75 (27/80)	26.73 (27/101)	34.38 (11/32)	38.89 (14/36)	31.91 (15/47)	40.74 (11/27)
Clinical pregnancy rate (%)	46.22 (348/753)	53.66 (22/41)	37.04 (20/54)	56.25 (9/16)	61.11 (11/18)	56.52 (13/23)	64.29 (9/14)
Early miscarriage rate (%)	2.87 (10/348)	0	10 (2/20)*	0	0	23.08 (3/13)^a^*	44.44 (4/9)*

^a^*P* value < 0.05, difference between chromosomal polymorphisms group and control group

^b^*P* value < 0.05, difference between female carriers and male carriers

*PN* pronuclei

*Fisher’s exact test

All continuous variables are expressed as mean ± SD

## Discussion

Until now, few reports have paid particular attention to the effects of female chromosomal polymorphisms on infertility, and no previous reports have stratified infertility according to the specific type of chromosomal heteromorphism on outcomes of IVF-embryo transfer. Our study firstly subdivided the chromosomal polymorphism infertility group into three groups (1/9/16qh^+/−^ group, D/G group, and inv(9) group) to investigate the association between detailed chromosomal polymorphism types and IVF/ICSI outcomes.

### 1/9/16qh^+/−^

It is common in chromosome polymorphism variations that the length of the secondary constriction in the long arm of chromosomes 1, 9, and 16 increases and decreases [31]. Both infertile males and females were found to frequently have a 9qh^+^ karyotype. It seems that the increase in highly repetitive DNA sequences in the distal chromosome segments may cause clinical symptoms. However, the structure and function of these duplicate DNA sequences in chromosomes 1, 9, and 16 remain unknown. In the current study, the results showed that for the 1/9/16qh^+/−^ group, there were no significant differences on embryological and clinical outcomes compared with those in the control group, regardless of whether IVF or ICSI was utilized. Also, there is no significant difference in embryological and clinical outcomes between different gender carriers. It appears that 1/9/16qh^+/−^ has no apparent adverse effect on the clinical outcomes, neither males nor females, ICSI or IVF, which is consistent with the current study.

### Variations in chromosome 9

The inversion of chromosome 9 heterochromatin is an observable structural difference between human karyotypes and chimpanzee karyotypes [32]. The mechanisms of the origin of inv(9) are highly complex [33]. Recently, the DNA sequencing and analysis of human chromosome 9 showed that it contains the largest autosomal block of heterochromatin and is highly structurally polymorphic and heteromorphic in 6–8% of humans. Several authors have suggested possible associations between the inv(9) and specific clinical observations, such as diagnosis of schizophrenia [34, 35], an increased risk of offspring with Down syndrome [8], and particularly, the occurrence of higher incidence of intrauterine fetal death [36]. However, the mechanism of the inv(9)’s effects on fertility has not been fully characterized.

In the present study, we found that inv(9) patients had a lower 2PN cleavage rate than that in the control patients when utilized IVF insemination but had no significantly difference when utilized ICSI. In addition, they had a higher early miscarriage rate both in the ICSI and IVF groups. We explored the role of gender on IVF/ICSI outcomes, and the results indicated that the 2PN cleavage rate of female inv(9) carriers was significantly lower than that in the control or male carrying patients. The role of inv(9) in human infertility remains unclear. Some authors have proposed that during meiosis, the inversion itself can interfere with the pairing of homologous chromosomes; this mechanism of recombination aneusomy is well described for some types of pericentric inversions [37]. Inv(9) has also been suggested to have some interchromosomal effects that lead to a higher incidence of mitotic disturbances, which is likely associated with aneuploidies, such as trisomy 21 [38]. This observation is in agreement with our findings that the cleavage rate of inv(9) patients is lower than that in the control patients. Šípek et al. compared inv(9) carriers and control subjects for each sex separately and found a statistically significantly higher incidence of heterochromatin variants (including the group of variants on chromosome 9) in females, but not in males, with idiopathic reproductive failure [39].This partially supported our findings that inv(9) in females leads to a lower cleavage rate and higher early miscarriage rate. Conversely, the study of Liang et al. indicated that the female carrier group had a higher normal fertilization rate and higher utilization rate than the male carrier group, which showed a tendency of better prognosis for the female carrier group [40]. This controversial results needs to be confirmed by a larger study. The possible gender-dependent differences in the potential meiotic mechanisms remain to be clarified.

Another finding of our study was that inv(9) leads to an increasing rate of early miscarriage Recently, Merrion et al. investigated the unbalanced chromosome rearrangement rate from inv(9) patients who underwent IVF with preimplantation genetic testing for structural rearrangements (PGT-SR) and found that the chromosome 9 pericentric inversions did not result in unbalanced structural rearrangements in day 5/6 embryo samples [41]. It was also reported that some genes located in 9p13, such as *Talin1* and *MELK*, might be related to early embryo implantation potential and/or endometrial receptivity [42], which help us inferring that we should consider the possibility of implantation potential and endometrial receptivity impacts of inv(9). Also, it could be due to the potential capability of eggs in repairing sperm-derived defects but not vice versa. The role of inv(9) in human infertility remains unclear, and the clinical importance of any individual inv(9) in a specific clinical pathology may be challenging to determine.

### D/G

D/G chromosomes are common chromosome heteromorphisms that show increased heterochromatin at the chromosome telomere, with short variants at the NORs. For the human acro chromosomes, the metaphase NORs contain ribosomal genes, and these genes are clustered on the short arm stalks, exhibiting polymorphic variations. For D/G chromosomes, the heterochromatin located in centromeres plays an important role in spindle attachment, chromosome movement, meiotic pairing, and sister chromatid cohesion [43]. To date, no study has reported the relationship between D/G group chromosomal variations and reproductive outcomes.

Our study first revealed that the 2PN cleavage rate of D/G polymorphism carriers was significantly lower than that in the control patients undergoing IVF treatments, but not ICSI. Surprisingly, in the ICSI group, the clinical pregnancy rate and implantation rate of the D/G patients were significantly higher than those in the control patients. Furthermore, the fertilization rate and 2PN cleavage rate of the female carriers were significantly lower than those of the control and male carrying patients, but no significant difference was found for male carriers. These results indicate that D/G chromosome polymorphisms in females lead to a lower fertilization rate and cleavage rate but these polymorphisms in male carriers seem to have no adverse effects on reproductive outcomes. Chromatin variations in D/G regions can cause defects in kinetochore assembly and centromere function, cause difficulty in homologous chromosome pairing, have impacts on cell division, and finally affect gamete formation. This also was supported by our research results that female carriers in the D/G group have lower fertilization and cleavage rates.

Increasing evidence has confirmed that female reproductive disorders are closely associated with chromosomal polymorphisms [29]. However, Madon et al. [3] reported that male partners displayed chromosomal polymorphism variations that had no adverse effects on pregnancy rates, suggesting that chromosomal heteromorphism in infertile males may have no adverse effect on IVF/ICSI treatment, which is consistent with our findings. Besides, the clinical pregnancy rate and implantation rate of the D/G group were significantly higher than those of the control group in ICSI patients, while there is no significant difference in patients with IVF insemination, which indicates that ICSI insemination might be a better choice for D/G chromosomal polymorphism patients. Also, considering the limited sample size of the ICSI patients, further study is needed to confirm.

Based on our results, we conclude that the inv(9) and D/G groups of chromosomal polymorphisms play essential roles in female infertility, leading to a lower cleavage rate and inv(9) adversely increasing the early miscarriage rate after IVF treatment. For D/G groups, ICSI insemination may benefit patients who have a chance of clinical pregnancy. 1/9/16qh^+/−^ has no apparent adverse effect on the clinical outcomes. We should provide individual genetic counseling suggestions according to the polymorphism type.

One limitation of our study is that the number of heteromorphism carriers undergoing ICSI is insufficient. For more statistical power, further studies with larger sample sizes are needed. Another limitation is that our study did not eliminate the potential effects of polymorphism variations on male sperm formation. Furthermore, the chromosome analysis method in the present study had a banding resolution of 400–550 BPHS, which may have caused some potential variations that could not be distinguished from common polymorphism variations.
